# Hearing Parents’ Voices: Parental Refusal of Cochlear Implants and the Zone of Parental Discretion

**DOI:** 10.1007/s11673-021-10154-8

**Published:** 2021-12-16

**Authors:** Owen M. Bradfield

**Affiliations:** Law & Public Health Unit, Centre for Health Policy, Melbourne School of Population & Global Health, University of Melbourne, Melbourne, Australia

**Keywords:** Autonomy, Cochlear implants, Consent, Zone of parental discretion

## Abstract

It has been forty years since the first multi-channel cochlear implant was used in Australia. While heralded in the hearing world as one of the greatest inventions in modern medicine, not everyone reflects on this achievement with enthusiasm. For many people in the Deaf community, they see the cochlear implant as a tool that reinforces a social construct that pathologizes deafness and removes Deaf identity. In this paper, I set out the main arguments for and against cochlear implantation. While I conclude that, on balance, cochlear implants improve the well-being and broaden the open futures of deaf children, this does not justify mandating implants in circumstances where parents refuse them because this may compound unintended harms when society interferes in the parent-child relationship. For this reason, I argue that parental refusal of cochlear implantation falls within Gillam’s concept of the zone of parental discretion.

## Introduction

Cochlear implants are small electronic devices that bypass damaged nerve cells and transmit signals via the auditory nerve to the brain (National Institute on Deafness and Communication Disorders 2016). They provide an effective treatment for profound hearing loss in children of at least twelve months of age (Hyde, Punch, and Komesaroff 2010). In the United States, 98 per cent of newborns are screened for hearing loss and two per 1000 are found to be profoundly deaf (Connolly, Carron, and Roark 2005). Therefore, cochlear implants are widely regarded in the hearing world as one of the greatest inventions of modern medicine. However, they are controversial within the Deaf community (Kim et al. 2010). Some Deaf individuals reject deafness as a disability and, instead, celebrate deafness as a unique culture in which the disadvantages of deafness are socially constructed (Barnes 2014) and could be remedied if society adapted to deaf people’s needs by, for instance, making sign language compulsory for all.

In this paper (Kahane and Savulescu 2009), I will argue that, because cochlear implants improve the well-being of implanted children and maximize their future options, implantation of profoundly deaf children appears to be in their best interests, from a hearing world perspective. However, mandatory implantation by the state may cause harm to children because the success of cochlear implants inextricably depends on the child and their carers being deeply committed to extensive follow-up and rehabilitation. Implanting children with cochlear implants against the wishes of parents may also undermine the parent-child relationship that the state endeavours to nurture. Therefore, I will argue that parental refusal of cochlear implantation is morally permissible because it falls within the zone of parental discretion, outlined by Gillam (2010). I will limit discussion to the use of cochlear implants in prelingually deaf children who cannot provide consent or a meaningful view about their own treatment.

## Arguments for Implantation

I will begin by assessing some of the arguments in favour of cochlear implantation. According to Freiman (2018), parents have a moral obligation to improve their children’s overall well-being by helping them live happier, healthier, and more successful lives. For example:

*Imagine you can send your child to one of two schools. They are equal in all respects, except that school A will better prepare your child for college and the job market than school B … a parent would be doing something wrong (nothing else considered) if they chose the worse school … for their child.*

This invites several ethical questions. Is deafness adverse to children’s welfare and well-being? Does cochlear implantation improve the welfare and well-being of implanted children? Are parents doing something wrong if they do not implant their deaf child? Is the failure to implant a profoundly deaf child so harmful that the state should intervene and implant the child against parental wishes?

The scientific literature tells us that people with profound hearing loss experience higher rates of mental illness, isolation, unemployment, and incarceration (Levy 2002). Cochlear implantation can overcome some of these problems. Implants have been shown to be safe and effective for children with profound hearing loss. Ten years post implantation, children with cochlear implants achieve hearing performance and spoken language acquisition comparable to hearing children (Peixoto et al. 2013). By high school, 75 per cent of implanted children are in mainstream education, with only 5 per cent requiring full time educational support in adapted educational settings (Geers, Tobey, and Moog 2011). The best results are achieved if profoundly deaf children are implanted at an early age. Implanting children before eighteen months of age results in superior speech perception and spoken language development (Kulkarni et al. 2018) because children experience auditory stimulation earlier when the developing auditory system is most plastic (Nicholas and Geers 2006). If children are implanted after thirty-six months of age, hearing and speech outcomes are compromised (Geers et al. 2017). When compared to acoustic hearing aids or non-technological support, cochlear implants improve linguistic, cognitive, emotional, and social development, quality of life, academic achievement, and employment prospects (Raine, Craddock, and Lutman 2010).

The evidence therefore suggests that cochlear implants may improve the well-being of those implanted in a predominantly hearing world. If we agree with Freiman that parents have a moral obligation to do the best for their children, and if it best for children be implanted, then it follows that parents may also have a moral obligation to implant their deaf child. However, there are shortcomings with this argument. First, much of the disadvantage experienced by the Deaf community is rooted in how society is structured and that this could be overcome through better supports and services. Second, many of the purported advantages with implantation are based on a hearing world perspective. Countering these shortcomings, Savulescu (Savulescu and Kahane 2011) asserts that the disadvantages of deafness transcend social prejudice or injustice. He believes that deafness makes people intrinsically worse off because it reduces the inherent goodness of a world replete with sound, music, and human voices. He argues that it is disingenuous to suggest that “the failure of a deaf person to hear the roar of an approaching tiger is the result of social construction.” He goes on to argue that failing to implant a profoundly deaf child amounts to child neglect (Savulescu and Kahane 2011), particularly given the improvements to cochlear technology and the reduction in risks.

The problem with Savulescu’s example is that only a hearing person who has experienced a world with sound can attach value to it. Moreover, there is evidence that deaf people may adapt to their deafness by developing better visual acuity (Shiell, Champoux, and Zatorre 2014). Similarly, the increasing use of assistive communication devices that convert speech to text and *vice versa* can overcome some of the problems faced by Deaf people in a hearing world. Devices can also connect to doorbells, telephones, or alarms and emit flashing lights that alert users (National Institute on Deafness and other Communication Disorders 2019). Therefore, it seems that while there are clearly disadvantages to being deaf in a hearing-centric world, the degree of harm caused by failing to implant a child is insufficiently serious to justify labelling it neglectful.

Another argument advanced in favour of cochlear implantation is that, in addition to maximizing their child’s well-being, it also fulfils their child’s “right to an open future” (Feinberg 1980). From the moment a child is born, her parents make choices that shape her future, such as choosing her religion or education (Levy 2002). The right to an open future can be violated when parents make decisions that constrain the possibilities available to their child as an adult. Therefore, parents should save these extant rights that children cannot assert as children until they reach adulthood. For example, Feinberg argues that Amish communities ought not prevent their children from attending school because it confines them to a life of illiteracy and manual labour. This is not only detrimental to society but also dramatically truncates the future options available to those children. By analogy, the argument is that parents who refuse to implant their deaf child would also be limiting their child’s open future. By confining the child to a world of deafness without spoken language, the future social, vocational, and educational possibilities available to the child may be restricted.

However, a future without hearing may not be so harmful to a profoundly deaf child that the state is justified in intervening to compulsorily implant that child against the sincere wishes of the child’s parent(s), who no doubt love their child and are doing their best to raise their child as best they can in a complex world.

## Arguments Against Implantation

### Preserving Deaf Culture

One of the strongest arguments against cochlear implantation is that it poses an existential threat to the Deaf community (Sparrow 2010). Many Deaf people reject deafness as a disability (Drolsbaugh 2008) and see themselves as members of a proud linguistic minority with a distinct language and culture. Deaf culture stems from a communication identity that binds the hearing impaired together in ways akin to other cultural groups (Weisleder 2012). This bond is unique, affirming, and mutually-supportive. The same cannot be said of the visually impaired as there is no recognized “Blind culture.” In part, this may be because sign language is seen as a distinct language, rather than a surrogate for spoken words, like Braille. Not surprisingly, members of the Deaf community perceive cochlear implants as reinforcing a social construct that seeks to “fix” a defect and expunge Deaf identity (Mauldin 2014). To them, inserting a cochlear implant is as deeply troubling as trying to change an individual’s skin colour (Kermit 2009). In addition to its intrinsic value, Deaf culture and diversity is instrumentally valuable because it affords identity and belonging to Deaf individuals, including those for whom cochlear implantation was either unavailable or unsuccessful. For parents to have genuine choices about the trajectory of their children’s lives, there must be more than one type of life and community available to them (Mill 1975).

Davis (1997) argues that when faced with a choice between maximizing a child’s future options and preserving a group’s culture, parents ought to choose to maximize their children’s future because, to deny these opportunities on the basis of artificial “theories of segregation” ignores the real possibility that children with maximized open futures who have been implanted can still embrace Deaf identity and transmit Deaf culture to their own children, even if they can hear. On this reasoning, concerns about the impact of implantation on the future of Deaf culture and identity is misplaced and should surrender to a paramount concern for the present and future welfare and open future of the deaf child, who may very well choose to participate in, and perpetuate, Deaf culture even as an implanted adult. This is particularly important when we consider that 90 per cent of deaf children are born to hearing parents who may not themselves identify with Deaf culture and may want their children to live in the hearing world (Pray and Jordan 2010). However, even if the preservation of Deaf culture is an insufficiently compelling reason not to implant profoundly deaf children, the existence of a Deaf culture that espouses and celebrates a Deaf identity helps to ameliorate the disadvantages that a deaf child may encounter later in life. For example, research shows that the early teaching of sign language as soon as a child’s deafness is detected can result in superior educational and vocational outcomes (Humphries et al. 2017). The Deaf community has been vocal in ensuring that the parents of deaf children are appropriately educated.

### Cochlear Implantation Undermines the Child’s Future Autonomy as an Adult

This argument asserts that prelingually deaf children are too young to be moral agents, so decisions that impact the child’s future linguistic and cultural identity should be deferred until the child can consent. In other words, deaf children should be allowed to grow up without implants so they can consent to cochlear implantation once they have been immersed in Deaf culture and once they have acquired the Deaf perspective (Bernicchia-Freeman 2018). However, there are also weaknesses with this argument.

The scientific literature informs us that, the earlier a prelingually deaf child receives an implant, the better chance they have of adapting successfully to the device (Marschark et al. 2018). Therefore, while deferring cochlear implantation until a child is of sufficient maturity to consent respects their future autonomy, it also reduces the chances of success. Admittedly, cochlear implantation is not a life-saving procedure. It involves drilling into the skull and carries intra-operative and post-operative risks, including infection, malfunction, neurological impairment, tinnitus, and poor auditory outcome. Indeed, it is because of these risks that some argue that cochlear implantation should be deferred until the child reaches adulthood and can freely choose to accept these risks (Bernicchia-Freeman 2018). However, cochlear implantation is no different to other treatments commonly undertaken in children, such as corrective eye surgery, where the benefit of these procedures can only be realized when performed during childhood. The case of cochlear implantation does not appear morally exceptional to analogous situations where parents must balance treatment risks and benefits and does not appear to provide a basis for arguing that it be deferred until adulthood.

### Cochlear Implantation Denies Deaf Children Their Birthright to be Deaf

A third argument against cochlear implantation of prelingually deaf children is that it violates a deaf child’s linguistic right to primarily use sign language (Murray 2015). The problem with this argument is that there appears to be no philosophical or empirical basis to support the assumption that deaf children should learn sign language before spoken language. Indeed, the morally relevant issue here is not whether a child has a right to acquire one language before another (or even whether one language is preferable to another) but about the right of every child to realize their full potential (Corker 1998). Implantation broadens, rather than restricts, the languages available to a child. If a child is implanted prelingually, they have the potential to grow up learning both sign and spoken languages. However, not implanting a deaf child confines them to the Deaf world:

[O]ne needs to think seriously about the limited opportunities that exist for even the most positively acculturated Deaf person. Marriage partners, conversation partners, vocations, and avocations are severely limited. Yes, one can think of cultural minorities about whom the same could be said—e.g., the Amish or very Orthodox Jews—but these children can change their minds as adults and a significant percentage do so*.* (Davis 1997)

This leads to the conclusion that cochlear implantation maximizes a child’s future options and does not limit them to either the hearing world or the Deaf world. It maximizes the child’s future life choices and enhances the child’s future autonomy, because the child can then decide which languages or cultures to embrace as an adult.

### Costs of Cochlear Implantation

Cost is also a morally relevant factor to consider when deciding if parents have a moral obligation to implant their deaf child in any given situation. In countries without universal healthcare arrangements, principally the United States, cochlear implantation may impose an insurmountable economic burden on uninsured parents. The cost of implantation alone ranges from US$30,000 to US$50,000, excluding additional costs associated with rehabilitation and replacement of parts (Nunez 2020). Where the costs of implantation are prohibitively expensive, parents should not have a moral obligation to implant their children because such a requirement would be overly demanding and would come at great cost to themselves and may, ultimately, deprive their child in other ways if the family is left destitute (Giubilini and Savulescu 2019). From an ethical perspective, there should be a limit to the kind of risks imposed on parents for the sake of their child’s benefit.

However, the United States is unique among OECD countries in not providing universal healthcare and, therefore, systematically discriminating against poor children born deaf. However, in other developed countries with nationalized health systems, cochlear implantation and associated treatments are freely provided by the state. In fact, a recent cost-benefit analysis in the Netherlands showed that the direct healthcare costs associated with cochlear implantation are offset by reducing educational and productivity costs (Neve et al. 2021). This provides a powerful rebuttal against arguments that the costs to taxpayers of cochlear implantation do not justify their use.

## Costs of Intervening

Even if, on balance, cochlear implantation is better for profoundly deaf children than not implanting them, this does not automatically mean that the state should forcibly implant a profoundly deaf child against the strong wishes of her parents. The United States Constitution protects a parent’s privacy right in child-rearing and recognizes that parents possess fundamental liberty interests in the care, custody, and management of their children, which includes the right to make medical treatment decisions (Brusky 1995). Although courts have shown general deference to parental decisions regarding a child’s medical care, that right is not absolute and courts have intervened when a parental decision threatens the child’s life, health, or emotional well-being. For example, courts have compelled lifesaving blood transfusions despite the objections of parents acting on their Jehovah’s witness beliefs (Zimmerman 2009). Likewise, many countries have criminalized the exquisitely painful and sometimes lethal practice of female genital mutilation, even though it is a socially-sanctioned ritual within some traditions that confers upon females full integration into their community (Davis 1997). However, discrete acts or omissions that threaten life or inflict pain may be disanalogous to cochlear implantation, the success of which requires years of committed after-care.

A child’s environment and family support are crucial to a cochlear implant’s success (Kim et al. 2010). The care required following cochlear implant surgery is lengthy and emotionally and physically draining for implanted children and their families (Tucker 1997). If parents are not deeply committed to the mapping, education, psychological therapy, rehabilitation, and support programmes that follow, the odds of success may be reduced and the implant may be ineffective (Hyde, Punch, and Grimbeek 2011). This could be more harmful for children than not implanting at all because, once implanted, a cochlear implant cannot be removed and children may neither acquire spoken language nor sign language (Zimmerman 2009).

More importantly, implanting a deaf child born to Deaf parents may cause harm to the deaf child if allowing them to hear results in the Deaf family feeling estranged from their child. Christiansen and Leigh (2002) suggest that for a deaf child born to a hearing family, enabling the profoundly deaf child to participate in the hearing culture of the parents through cochlear implantation contributes to the well-being of the child by strengthening the parent-child relationship and their sense of belonging. Likewise, allowing a deaf child to remain deaf without a cochlear implant could allow the deaf child to develop stronger bonds with her Deaf parents and allow her to flourish and become a valued member of a supportive Deaf community. Deaf parents may consider that they would better parent a deaf child than a hearing child (Tuchler 2009), while implantation could corrupt a deaf child’s relationship with their parents.

The effects of cochlear implantation on a child’s language, culture, education, employment, and social milieu demands that parents’ values, needs, and desires are understood, even if they seem contrary to the child’s best interests. These views command compassion, not condemnation. The impact of altering a child’s cultural belongingness and identity through implantation should not be under-estimated. Compelling surgery for children whose parents object to cochlear implants may significantly interfere with the parent-child relationship that society strives to protect (Sparrow 2005). Assuming the parents’ beliefs are genuine and sincere, quashing these genuine cultural values via judicial decree could cause more harm to the child and her family by undermining the rights of all parents to determine the best interests of their children.

While there are undoubtedly harms from not implanting the child, compulsory implantation may inadvertently compound pre-existing disadvantage. Indeed, there is also a moral case for compulsory sign language education in mainstream schools because it may benefit all children, especially deaf children (Bowman-Smart et al. 2019). Likewise, greater involvement of the Deaf community in cochlear transplantation programmes may also better serve the long-term interests of the deaf child, her parents, and the Deaf community.

## The Zone of Parental Discretion

I have outlined arguments for and against cochlear implantation and highlighted some of the putative harms if parental refusal is ignored and overridden. In this final section, I will apply Gillam’s practical approach to balancing competing benefits and harms to determine whether parental refusal of cochlear implantation legitimately occupies the “zone of parental discretion” and is therefore morally permissible (Gillam 2016).

Resolving disputes between parents and doctors about paediatric treatment is challenging. Historically, a *best interests* approach has been adopted. However, the main problem with this approach is that it often results in moral criticism of any decision that is not the “absolute best” for the child (Buchanan and Brock 1998), even though this may sometimes be unrealistic or unachievable. Later, Diekema redirected our attention towards the Harm Principle. According to this principle, our ethical attention should be focussed on avoiding harm to the child, not on pursuing what is optimal (Diekema 2004). This recognizes that many decisions may be ethically acceptable, even if they are not optimal, provided they do not cause harm.

According to Gillam, the zone of parental discretion refers to this “ethically protected space” where parental decisions about their child are “good enough.” In other words, while they may not fully meet a child’s interests and may therefore fall short of being optimal, they are not so bad as to be harmful. Therefore, they extend from the best decisions at the top, to just above harmful decisions at the bottom. Gillam proposes a two-stage process for determining whether a parental decision falls within the zone of parental discretion. The first step is to determine whether upholding the parental decision will cause harm. Diekema requires this harm to be significant, serious, imminent, and preventable. The second step is to balance this against harm that may result from overriding parental refusal.

In this paper, I have shown that not implanting a cochlear implant is clearly sub-optimal for a profoundly deaf child in a predominantly hearing world because it can lead to social and economic disadvantage. However, I have also shown that these risks can be off set, at least in part, by parental love and commitment, acceptance into Deaf culture, and the use of assistive technologies. Therefore, it is equivocal whether refusing to implant a profoundly deaf child is overtly harmful such that overriding parental refusal is morally justified. Indeed, even if it is harmful, overriding parental choice undoubtedly creates risks of harm, including: weakening the parent-child relationship; misunderstanding the social, psychological, and emotional needs of the child that parents are best placed to appreciate; and subjecting the child to medical treatments that may be unsuccessful (and more harmful than no treatment) if parents cannot commit to the process.

At length, while failing to implant a profoundly deaf child appears to be an ethically sub-optimal choice from a hearing world perspective, implanting that child against the strong opposition of parents may be far more damaging. Therefore, it seems, when applying Gillam’s concept of the zone of parental discretion to cochlear implantation, parental refusal ought to be tolerated. In other words, if intervening may cause more harm than not intervening, we ought not to intervene, as this is also most consistent with the time-honoured principle of *primum non nocere* (Gillon 1985).

## Conclusion

As cochlear implant technology improves, more children will be implanted. From a hearing perspective, implants gift children the joy of sound, improve well-being, happiness, educational attainment and employability. However, while some people argue that the Deaf community should not impose its opposition to cochlear implants on parents and children with profound hearing loss, the same is true of the hearing world. If a child was born without legs, society would object if the child’s parents refused prostheses for their child. So too, society would strongly object if parents also refused to implant their deaf child (Zitter 1994). However, in a successful modern pluralistic society, we seek to celebrate and integrate disparate religious, ethnic, and racial traditions (Nunes 2001). Contempt should cede to common understanding in promoting the well-being of the child. While declining to implant a deaf child may reduce a child’s well-being from a hearing perspective, forcibly implanting a child against the vehement wishes of parents may flagrantly unleash harm. Most importantly, society would be disadvantaged if it lost a vibrant and strong Deaf community. Cochlear implants do not always work. If Deaf culture were to be forgotten because of hostility towards its restrictive views, there will be fewer lifestyle choices available to those for whom cochlear implants are unavailable or unsuccessful. Indeed, implanted children also lose the opportunity to grow up embracing a Deaf identity and passing on its irreplaceable culture to their own children. In all likelihood, the very survival of Deaf culture depends on it embracing cochlear implants and the paramountcy of the welfare and interests of the deaf child. Over the last forty years, we have come a long way in our understanding of cochlear implant technology. So too have we evolved in our appreciation of what it means to have a disability and to be a member of a linguistic minority. For these reasons, parental opposition to cochlear implantation ought to occupy the zone of parental discretion and is therefore morally permissible.
